# SARS-CoV-2 Effects on Respiratory and Neurological Systems: Morphological Findings and Gene Expression in K18-hACE2 Mice Model

**DOI:** 10.3390/microorganisms14040852

**Published:** 2026-04-10

**Authors:** Ana Luisa Teixeira de Almeida, Andréa Marques Vieira da Silva, Mariana Mello e Souza, Miguel Pires Medeiros Diniz Rodrigues, Felipe Soares Coelho, Lorenna Carvalho da Rosa, Tamiris Azamor, Carolina Baeta Salvador Várady, Bruno Jorge Duque da Silva, Alex Costa de Almeida, Renata Tourinho Santos, Rodrigo Müller, Rafael Braga Gonçalves, Ana Paula Dinis Ano Bom, Debora Ferreira Barreto-Vieira

**Affiliations:** 1 Laboratory of Viral Morphology and Morphogenesis, Oswaldo Cruz Institute, Oswaldo Cruz Foundation—Fiocruz, Rio de Janeiro 21040-900, RJ, Brazil; 2 Laboratory of Immunological Technology, Department of Experimental and Preclinical Development, Bio-Manguinhos, Oswaldo Cruz Foundation—Fiocruz, Rio de Janeiro 21040-900, RJ, Brazil; amarques@bio.fiocruz.br (A.M.V.d.S.); miguel.rodrigues@bio.fiocruz.br (M.P.M.D.R.); felipe.coelho@bio.fiocruz.br (F.S.C.); lorenna.rosa@bio.fiocruz.br (L.C.d.R.); tamiris.azamor@bio.fiocruz.br (T.A.); adinis@bio.fiocruz.br (A.P.D.A.B.); 3 Laboratory of Preclinical Trials, Department of Experimental and Preclinical Development, Bio-Manguinhos, Oswaldo Cruz Foundation—Fiocruz, Rio de Janeiro 21040-900, RJ, Brazil; mariana.msouza@bio.fiocruz.br (M.M.e.S.); carolina.varady@bio.fiocruz.br (C.B.S.V.); bruno.duque@bio.fiocruz.br (B.J.D.d.S.); alex.costa@bio.fiocruz.br (A.C.d.A.); rmuller@bio.fiocruz.br (R.M.); 4 Laboratory of Virological Technology, Department of Experimental and Preclinical Development, Bio-Manguinhos, Oswaldo Cruz Foundation—Fiocruz, Rio de Janeiro 21040-900, RJ, Brazil; renata.tourinho@bio.fiocruz.br; 5 Department of Biochemistry, Biomedical Institute, Federal University of the State of Rio de Janeiro, Rio de Janeiro 20211-040, RJ, Brazil; rafael.braga@unirio.br

**Keywords:** SARS-CoV-2, K18-hACE2 mice, histopathology, transmission electron microscopy, gene expression

## Abstract

The COVID-19 pandemic, caused by severe acute respiratory syndrome coronavirus 2 (SARS-CoV-2), has revealed a complex interplay between respiratory and neurological manifestations. This study utilized K18-hACE2 transgenic mice to investigate the morphological, ultrastructural, and transcriptomic changes induced by SARS-CoV-2 infection in both lungs and brain tissues. Histopathological analysis at seven days post-infection revealed significant pulmonary damage characterized by interstitial pneumonia, alveolar septal thickening, with a marked inflammatory infiltrate predominantly consisting of neutrophils and lymphocytes, and an abnormal profile of type II pneumocytes. Concurrently, in the brain, we observed vasculitis, gliosis, and edema, indicating an inflammatory response and vascular compromise that can disturb the blood–brain barrier. In addition, gene expression in lung tissue presented increased *CCL2*, *IL10*, and *GDDA45D* in infected mice and the downregulation of proinflammatory genes. However, in brain tissue, the increased expression of *CCL2*, *CASP1*, *IL6*, *IFNB1*, and *GDDA45G* inflammatory genes was observed in infected K18-hACE2 mice.

## 1. Introduction

The emergence of severe acute respiratory syndrome coronavirus 2 (SARS-CoV-2) resulted in the coronavirus disease 2019 (COVID-19) pandemic. An unpredictable scenario unfolded in global health, associated with a wide clinical spectrum ranging from asymptomatic infections to fatal cases. When symptomatic, fever, cough, fatigue, and shortness of breath are the main manifestations [1]. Although respiratory involvement remains one of the most common in overall cases, significant outcomes related to the nervous system have been reported [2]. Neurologic outcomes include headache, dizziness, hyposmia, hypogeusia and, in severe cases, seizures, strokes, and ataxia may occur [3].

The COVID-19 pandemic culminated in millions of deaths and still resonates in current consequences such as the cocirculation of multiple SARS-CoV-2 variants and long-lasting effects in patients [4]. Long COVID refers to the syndrome characterized by the onset of symptoms that can endure for years in patients, even after the convalescence phase of COVID-19 [5]. It represents an upcoming worldwide emergency with predominantly neurological sequelae, including cerebrovascular, cognition, memory, sensory, and mental health disorders [6]. The cardiovascular and musculoskeletal systems may be affected and, as with regular COVID-19, the respiratory function usually shows different degrees of impairment [7].

The advent of effective vaccines and therapeutics and the conducting of studies about SARS-CoV-2 are strongly linked to the development of animal models susceptible to the infection [8]. Certain aspects in the interaction between the virus and the host are essential to enable appropriate experimental infections, such as the virus’s entry into permissive cells, which represents a crucial step in the resulting pathogenesis [9]. The spike protein of SARS-CoV-2 binds to the host angiotensin-converting enzyme 2 (ACE2) protein for cell entry [10]. This interaction determines both susceptibility to infection and the severity of the disease [11,12].

However, the effectiveness of this interaction is species-specific, occurring more effectively in some groups of animals. For example, while SARS-CoV-2 binds adequately to human and hamster ACE2, in murine models the interaction between mouse-ACE2 and the SARS-CoV-2 spike protein is not as effective [13]. Consequently, strategies were devised to still utilize mice and adapt them to increase the affinity necessary for robust replication. Most studies have focused on transgenic mice expressing the human ACE2 protein under the control of the keratin 18 promoter (K18-hACE) [14].

The similar distribution of the ACE2 receptor in different organs of hamster, compared with human, may be extended to the observed resemblance in tissue damage and clinical outcomes in hamster models and human cases [15]. In contrast, K18-hACE2 mice exhibit atypical expressions of ACE2, with increased levels in the brain [16]. This expression likely facilitates neuroinvasion, and, along with secondary factors, contributes to the higher lethality observed in these experimental models [17].

More studies are needed towards the distribution of the ACE2 receptor comparatively in organs of K18-hACE mice and humans. Despite this, K18-hACE models present robust replication to assess weight loss, neuroinvasion following intranasal inoculation with SARS-CoV-2, and morphological changes in various organs that are consistent with human cases, making them excellent for studying severe forms of the disease [18,19].

Despite the multiorgan targeting potential of this viral infection, understanding the pathogenesis in the lungs and brain is an essential first step in addressing the many uncertainties related to the SARS-CoV-2 dynamics as these organs appear to have an intrinsic relationship throughout the infection. Common in human cases of COVID-19, some changes observed in the lungs, and consequently in respiratory function, such as atelectasis, shortness of breath, and acute respiratory distress, may be caused by neurological and neuromuscular damage [16].

In light of this, our aim in this study was to evaluate morphological patterns of SARS-CoV-2 infection of K18-hACE in these organs, from a histological approach to a refined look through transmission electron microscopy to assess ultrastructural aspects. Additionally, we evaluate the changes in gene expression of some markers’ proinflammatory and regulatory immune responses, as well as of oxidative stress genes related to the lung and brain functioning.

## 2. Materials and Methods

### 2.1. Experimental Design

A total of forty-two male and female K18-hACE2 mice, aged between 12 and 26 weeks, were utilized. The mice were separated into two groups: Group 1 (negative control) was inoculated with phosphate-buffered saline (PBS), while Group 2 was inoculated with 1 × 10^5^ SARS-CoV-2 Wuhan variant viral particles. The animals were anesthetized via inhalation using up to 2 to 3% isoflurane (dose-dependent) in an acrylic anesthesia chamber specifically designed for laboratory animals (INHALATION ANESTHESIA EQUIPMENT BY INFUSION—Bonther^®^, São Paulo, Brazil). After complete relaxation, the animals were kept in an upright position and inoculated intranasally with 10 µL (5 µL per nostril) using a 2–20 µL automatic pipette (Rainin^®^, Oakland, CA, USA).

All procedures involving animals were conducted in Animal Biosafety Level 3 at the Preclinical Testing Laboratory (LAEPC) of the Department of Experimental Development and Preclinical Research at Bio-Manguinhos/Fiocruz, Rio de Janeiro, Brazil. The experiment was previously approved by the Animal Ethics Committee (CEUA) of Fiocruz (license number LW-08/20, approved on 15 June 2020). The animals were provided by the Institute of Science and Technology in Biomodels (ICTB)/Fiocruz, Rio de Janeiro, Brazil, and housed at a density of four animals per cage, considering varying weights to ensure heterogeneity within each group. The airtight cages measured 36 cm in length, 17 cm in width, and 15 cm in height, and were set up in a ventilated microisolator rack system (ALESCO^®^, São Paulo, Brazil) using high negative pressure system (ALN2) with autoclavable wood shavings as substrate. Water and food were changed twice a week under unrestricted access conditions. The mice were maintained under a 12 h light/12 h dark cycle in a controlled environment with temperatures ranging from 18 to 24 °C, relative humidity between 40 to 70%, and a constant air exchange rate of 20 changes per hour.

The experimental laboratory has an Environmental Enrichment Program that includes a polysulfone igloo as a shelter, with cotton rolls, shredded paper, and cardboard being interspersed monthly as a form of environmental enrichment. Clinical evaluations of the animals were conducted by a veterinarian based on the ARRIVE guidelines, and humane endpoints were established based on the severity of clinical signs, such as piloerection, arched back, respiratory distress, locomotion difficulties, and ocular discharge. Euthanasia was performed through anesthesia with doses of 200 mg/kg of ketamine hydrochloride and 20 mg/kg of xylazine hydrochloride administered intramuscularly. After several minutes of anesthetic administration and the absence of palpebral and inter-digital reflexes tested by touch, sodium thiopental was administered intraperitoneally at a dose of 150 mg/kg. The health monitoring of the colony providing the animals was carried out quarterly based on a specific pathogen list, as recommended by the Federation for Laboratory Animal Science Associations (FELASA), with all animals included in this study considered free of specific pathogens (SPF).

### 2.2. Clinical Signs

The mice were evaluated from the first to the seventh day of the experiment, following the SARS-CoV-2 inoculation, or inoculation of PBS, for the negative control group mice. Throughout the study, several clinical signs and parameters were assessed, including piloerection, presence of an arched back, respiratory alterations, ocular discharge, and difficulty in locomotion or paralysis. On the seventh day post-infection, all mice were euthanized according to institutional ethical guidelines.

### 2.3. Brighfield Microscopy

The lung and brain tissue samples were dehydrated in increasing concentrations of ethanol, clarified in xylene, and embedded in paraffin. Subsequently, a Leica 2025 microtome (Leica, Wetzlar, Germany) was used to section the paraffin blocks. The slices, 5 µm thick, were then placed in glass slides, stained with hematoxylin and eosin (H&E), and finally analyzed using a bright field microscope (AxioHome, CarlZeiss, Oberkochen, Germany).

### 2.4. Histomorphometry

To assess quantitative data on morphological changes in pulmonary alveoli, a total of 41 histological slides were evaluated (negative control group = 21, infected group = 20). Each animal was represented by a histological slide, for which 20 images of different alveolar fields were captured at a 200× magnification with a camera coupled to a bright field microscope (AxioHome, CarlZeiss, Oberkochen, Germany). For each image, the thickness of 40 alveolar septa was quantified using the ImageJ software version 1.53t (NIH ImageJ, National Institutes of Health, Bethesda, MD, USA). With the data compiled, the normality of the distribution was evaluated, and Student’s *t*-test was performed, with *p* ≤ 0.05 considered statistically significant. Both tests and graphics were made with aid of GraphPad Prism 8.0.1 software (GraphPad Software Inc., La Jolla, San Diego, CA, USA).

### 2.5. Transmission Electron Microscopy

For transmission electron microscopy analysis, lung and brain samples were collected and fixed by immersion in 2.5% glutaraldehyde in sodium cacodylate buffer 0.2 M, pH 7.2 (Electron Microscopy Sciences, Hatfield, PA, USA) [20]. Subsequently, the samples were post-fixated in 1% buffered osmium tetroxide (Electron Microscopy Sciences, Hatfield, PA, USA), dehydrated in acetone, embedded in epoxy resin (Electron Microscopy Sciences, Hatfield, PA, USA), and polymerized at 60 °C over three days [21]. Ultrathin sections within 50 to 70 nm were obtained from the resin blocks. The sections were picked up using copper grids with 300 mesh and no coating and observed under the Hitachi HT 7800 (Hitachi, Tokyo, Japan) transmission electron microscope.

### 2.6. mRNA Quantification in Lung and Brain Tissue

For gene expression analysis, a portion of the lung and brain tissue was preserved using Invitrogen RNAlater^®^ (Thermo Fisher Scientific, Waltham, MA, USA) and stored as recommended by the manufacturer. From the extracted mRNA from lung and brain tissue of mice seven days post-infection using the RNeasy Plus Mini Kit (Qiagen, Venlo, The Netherlands), performed according to the manufacturer’s instructions, the cDNA synthesis from 500 ng of total RNA using the high-capacity cDNA reverse transcription kit (Applied Biosystems, Waltham, CA, USA). Quantitative PCR (qPCR) was carried out with SYBR^®^ Green master mix (Applied Biosystems, Waltham, CA, USA), 400 nM of each primer (forward and reverse), and 5 ng of each cDNA. The expression levels of the genes *IL1B*, *IL6*, *IL18*, *FTH1*, *NOX1*, *IL10*, *CASP1*, *GADD45G*, *IFNB1*, *TLR4*, *TLR9*, *CCL2*, and *CLEC5A* transcripts were quantified using house-keeping genes *B2M*, *GAPDH*, and *PPIA*. The cycling conditions used were 10 min at 95 °C, 15 s at 95 °C, and 4 min at 60 °C, for 45 cycles for DNA denaturation and amplification carried out using the QuantStudio Pro 7 Real-Time PCR System (Applied Biosystems, Waltham, CA, USA).

The mean PCR efficiency per amplicon was determined using LinRegPCR software version 2021.2 and used to calculate the initial target concentration (N0), expressed in arbitrary fluorescence units. Data were normalized by dividing the mean N0 of the target genes by the mean N0 of the reference genes. The results were expressed on a logarithmic scale. For statistical analysis, the Kolmogorov–Smirnov test was performed to identify the normality assumption of the data. Log2-normalized expression was compared among negative control group and infected mice group using Mann–Whitney test, with two-tailed significance levels of ≤0.01, 0.05, and 0.1 considered as “highly significant”, “significant”, and “suggestive”, respectively. Graphs and statistical analyses were performed using Prism software version 8.4.2 (GraphPad Software Inc., La Jolla, San Diego, CA, USA).

## 3. Results

### 3.1. Clinical Signs

In the control group, no clinical signs were observed throughout the experimental period. The animals exhibited normal behavior, without physiological alterations or signs of distress, including respiratory or motor abnormalities, or any other symptoms associated with infection. In contrast, in the infected group, all animals remained asymptomatic until the fourth day post-infection. However, from the fifth day onward, the first clinical signs began to appear, becoming progressively more evident in the following days. The most frequently observed symptoms included piloerection, hunched posture, lethargy, respiratory distress, and reduced spontaneous movement. Additionally, more severe signs, such as ocular discharge and locomotion difficulties, were detected in some individuals during the final days of evaluation. The progression of symptoms suggests an initial asymptomatic phase, followed by a rapid onset of clinical manifestations consistent with SARS-CoV-2 infection. This observation reinforces the potential of the K18-hACE2 mouse model for studies investigating the pathogenesis of COVID-19 and its systemic implications. The table below (Table 1) presents the frequency of clinical signs observed in infected mice on the fifth, sixth, and seventh days post-infection.

### 3.2. Lungs Histopathology

**Figure 1:**
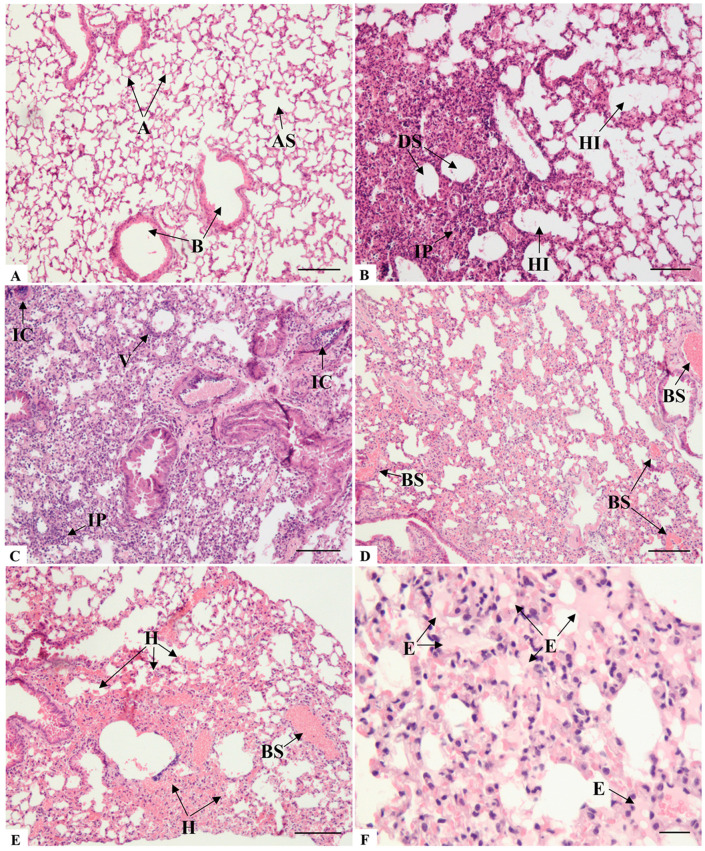
Histopathological analysis of lung sections of K18-hACE2 mice inoculated with PBS (**A**) and infected with SARS-CoV-2 (**B**–**F**). (**A**) A: Alveoli; AS: Alveolar sac; B: Bronchiole. Comparatively, note the difference between the alveolar architecture of non-infected and infected mice, particularly concerning the thickening of the alveolar septa in the infected group. (**B**) IP: Interstitial pneumonia; DS: Dead spaces; HI: Hyperinflation. (**C**) IC: Inflammatory cuffing; IP: Interstitial pneumonia; V: Vasculitis. (**D**) BS: Blood stasis; (**E**) BS: Blood stasis; H: Hemorrhage; (**F**) E: Edema. Bar: (**A**–**E**): 100 μm, (**F**): 50 μm.

The lung histological assessment of the control group mice (animals inoculated by intranasal route with PBS) revealed well-preserved bronchioles and alveolar sacs, as well as unobstructed alveoli with thin septa (Figure 1A). At seven days post-infection, the intranasal SARS-CoV-2 infection of K18-hACE2 mice induced several pulmonary pathologies. Analysis of the pulmonary architecture revealed primary alterations consistent with interstitial pneumonia, characterized by a predominantly lymphocytic and neutrophilic infiltrate (Figure 1B,C).

The spectrum of alveolar morphology varied between areas of the lung. Frequently, the regions of increased thickening or even complete consolidation of the alveolar wall, resulting in collapsed alveoli, were surrounded by zones with alveolar hyperinflation (Figure 1B). This mechanism serves as a compensatory measure to maintain respiratory function by preserving gas exchange in the adjacent alveolar spaces. However, many regions, due to hyperinflation, exhibited rupture and damage of the alveolar septa, consequently leading to the formation of dead spaces (Figure 1B), airway luminal areas that are no longer capable of facilitating gas exchange.

Hemodynamic changes also occurred, resulting in a significant obstructive pattern. Blood vessels of various calibers exhibited blood stasis (Figure 1D,E), and extravasation of proteinaceous content into the alveolar spaces (Figure 1F) was also observed, indicating congestion and increased vascular permeability. Despite all the vascular-related changes that could contribute to a hemorrhagic state, bleeding foci were few, and extensive hemorrhagic regions were rarely observed (Figure 1E).

**Figure 2:**
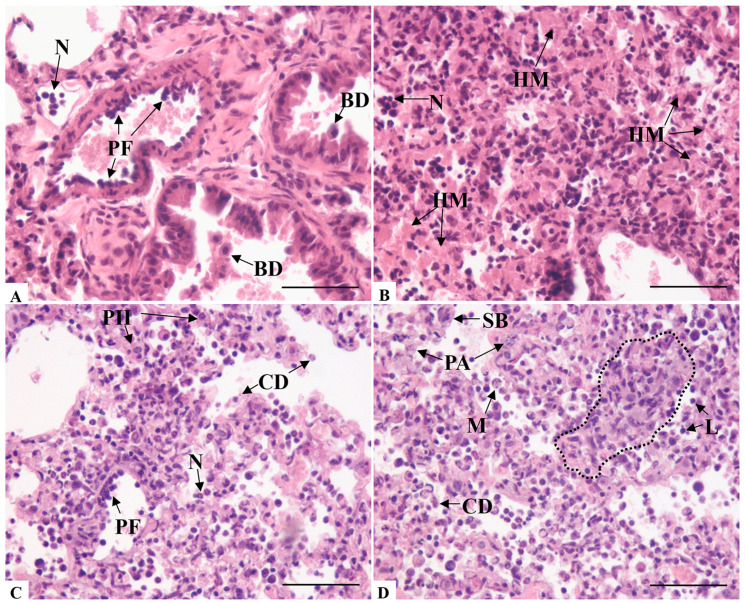
Histopathological analysis of lung sections of K18-hACE2 mice infected with SARS-CoV-2 (**A**–**D**). (**A**) BD: Cellular desquamation of bronchiole epithelial cells; PF: Perivascular inflammatory infiltrate; N: Neutrophilic infiltrate. (**B**) HM: Hyaline membrane-like structures; N: Neutrophilic infiltrate. (**C**,**D**) Note the hypercellularity profile, characterized by intense circulation of inflammatory cells within the luminal spaces and infiltration into the lung parenchyma. (**C**) CD: Cellular debris; N: Neutrophilic infiltrate; PF: Perivascular inflammatory infiltrate; PII: Type II pneumocyte. (**D**) M: Macrophage; CD: Cellular debris; L: Lymphocytic infiltrate; PA: Type II pneumocyte atypia. Note that in addition to highly vacuolated and hyperthrophic type II pneumocytes, area of hyperplasia in the parenchyma (dashed area); SB: Sintitial body. Bar: (**A**,**B**): 50 μm, (**C**,**D**): 100 μm.

Perivascular inflammatory infiltrates were consistently detected, along with frequent circulation within the vascular lumen (Figure 2A), even occluding it (Figure 1C). Alterations in endothelial cells were also noted, including inflammation of the vascular wall, resulting in the formation of multiple endothelial layers (Figure 2A). The intense infiltration of inflammatory cells into the alveolar parenchyma paired with the formation of hyaline membrane-like structures (Figure 2B).

The most significant alteration in the bronchioles was cellular desquamation (Figure 2A). In contrast, the alveolar structure exhibited a generalized hypercellularity profile, greatly influenced by type II pneumocytes, which played a critical role in altering alveolar structure. Certain pleomorphism was observed among these cells that frequently appeared abnormal with size atypia or pronounced cytoplasmic vacuolization, resulting in a foamy cytoplasmic appearance (Figure 2C,D).

### 3.3. Lungs Histomorphometry

**Figure 3:**
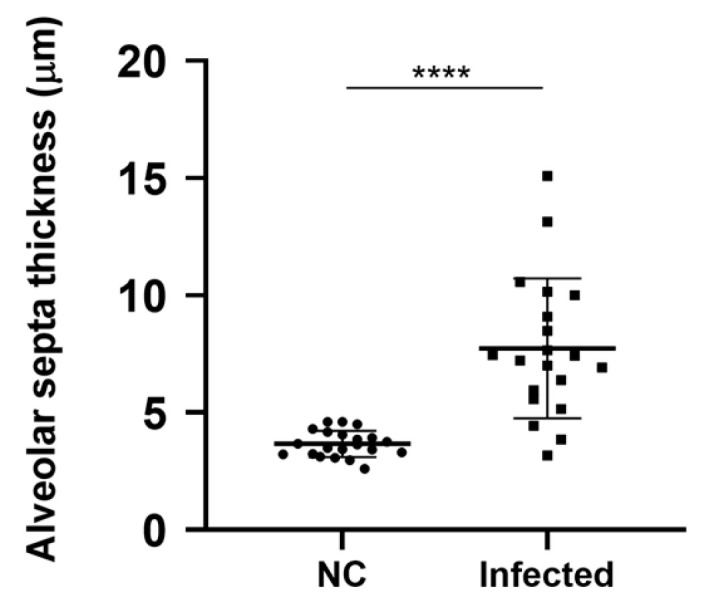
Morphometric analysis of alveolar septa thickness of non-infected and infected K18-hACE2 mice. NC: Negative control group. Student’s *t*-test **** *p* < 0.0001.

Given that changes in alveolar morphology associated with interstitial pneumonia were significant in the initial histopathological evaluation, we conducted a quantitative analysis of alveolar septal thickness. Our analysis showed that the mean thickness of alveolar septa (mean = 7.740 µm) observed in infected K18-hACE mice was two times greater than in the negative control group (mean = 3.668 µm). The difference between the two groups was statistically significant (*p* < 0.0001) (Figure 3). The infected animals showed a significant range in their values, with the lowest and highest values of 3.167 µm and 15.091 µm, respectively.

The previous alterations cited in the histopathological analysis, specifically the interstitial pneumonia and type II pneumocytes hyperplasia, were corroborated by the quantitative analysis of the alveolar septa thickness. The broad range of values found among infected mice were related to the arrangement of these alterations. Since all animals exhibited areas of notable pneumonia, the majority of these were focal, leading to less pronounced final mean values for the thickness of the alveolar septa. Nonetheless, values were also recorded that were as much as three times greater than those observed in the control group mice, indicating instances of diffuse alveolar damage.

### 3.4. Lungs Ultrastructure

**Figure 4:**
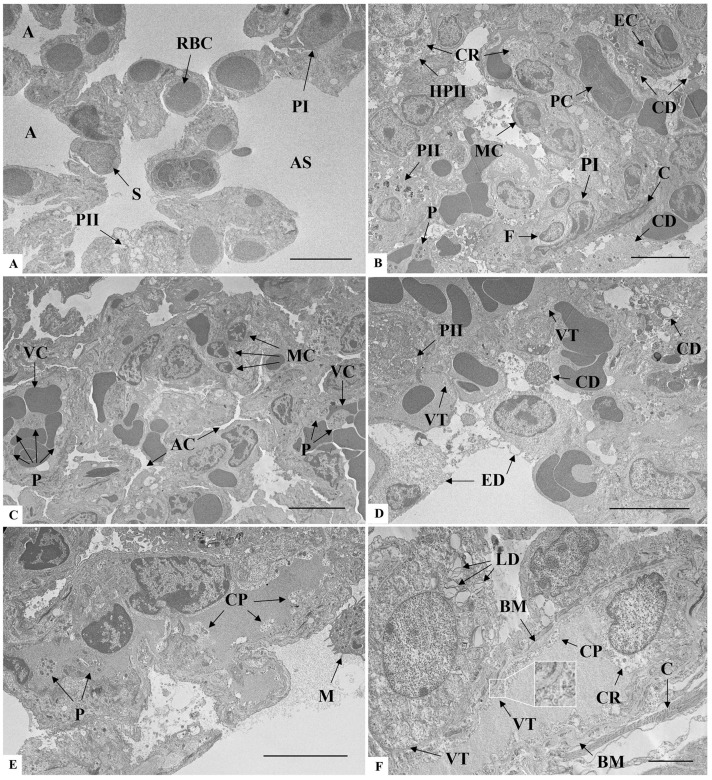
Ultrastructural analysis of lung sections of K18-hACE2 mice inoculated with PBS (**A**) and infected with SARS-CoV-2 (**B**–**F**). (**A**) A: Alveoli; AS: Alveolar sac; PI: Type I pneumocyte; RBC: Red blood cell; S: Alveolar septa. (**B**) C: Collagen; CD: Cellular debris; CR: Cytoplasmic rarefaction; F: Fibroblast; HPII: Hypertrophic type II pneumocyte; MC: Mononuclear inflammatory cell; P: Platelet; PII: Type II pneumocyte. (**C**) AC: Alveolar compression; P: Platelet; VC: Vascular congestion. (**D**) CD: Cellular debris; ED: Epithelial cell desquamation; PII: Type II pneumocyte; VT: Vesicle trafficking. (**E**) M: Macrophage; CP: Cytoplasmic protrusion; P: Platelet. (**F**) BM: Basal membrane thickening; C: Collagen; CP: Cytoplasmic protrusion; CR: Cytoplasmic rarefaction; LD: Lamellar bodies degeneration of type II pneumocyte; VT: Vesicle trafficking. Bar: (**A**–**E**) 5 μm, (**F**) 2 μm.

The main ultrastructural changes observed in infected mice included the following: infiltration of mononuclear (lymphocytes) and polymorphonuclear (neutrophils) cells into the parenchyma (Figure 4B,C) resulting in a thickening of the alveolar septa (Figure 4B,C) and alveolar collapse (Figure 4C). Vascular involvement was represented by congestion (Figure 4C), platelet recruitment (Figure 4B,C,E), basal membrane thickening, and cytoplasmic vesicle trafficking and protrusions in endothelial cells (Figure 4E,F). The aforementioned changes were not observed in the animals of the negative control group (Figure 4A).

**Figure 5:**
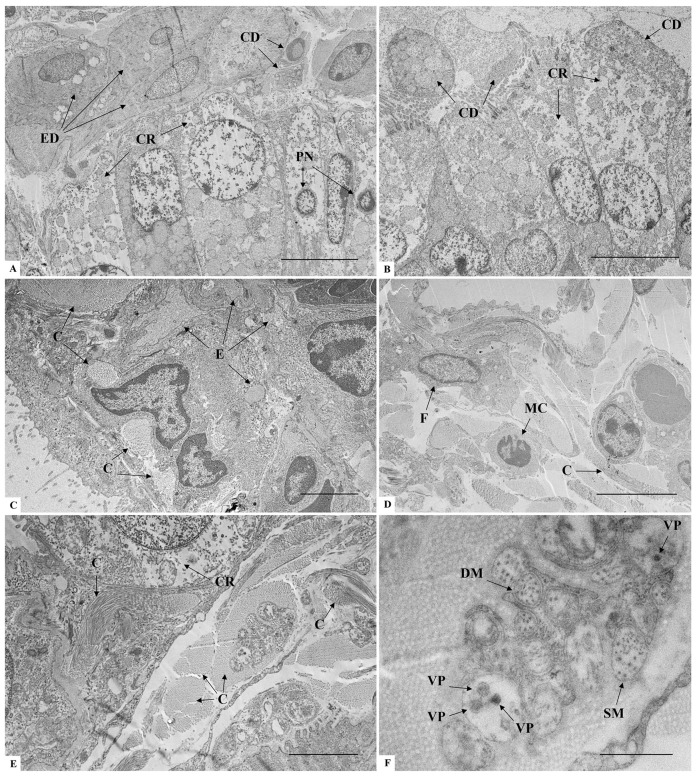
Ultrastructural analysis of lung sections of K18-hACE2 mice infected with SARS-CoV-2 (**A**–**F**). (**A**) CD: Cellular debris; CR: Cytoplasmic rarefaction in club cells of alveolar bronchiole; ED: Epithelial cells desquamation; PN: Pycnotic nuclei in club cells. (**B**) CD: Cellular debris; CR: Cytoplasmic rarefaction. (**C**) C: Collagen; E: Edema. (**D**) C: Collagen; F: Fibroblast; MC: Mononuclear inflammatory cell. (**E**) C: Collagen; CR: Cytoplasmic rarefaction. (**F**) Inset of Figure 5E. DM: Double-membrane vesicle; SM: Single-membrane vesicle; VP: Virus-like particle (mean diameter: 78 nm). Bar: (**A**,**B**,**D**) 5 μm, (**C**,**E**) 2 μm, (**F**) 500 nm.

The main change regarding the bronchiolar structure was the cell degeneration, represented by either the cytoplasmic rarefaction, pycnotic nuclei, or cellular debris (Figure 5A,B). Epithelial desquamation also occurred into the alveolar space (Figure 4A). Collagen deposition was extremely relevant, occupying intercellular spaces in the alveolar septa and the alveolar lumen (Figure 5B,E). Structures consistent with the characteristics of SARS-CoV-2 viral particles were observed in vesicles compartments surrounded by collagen (Figure 5E,F).

### 3.5. Brain Histopathology

**Figure 6:**
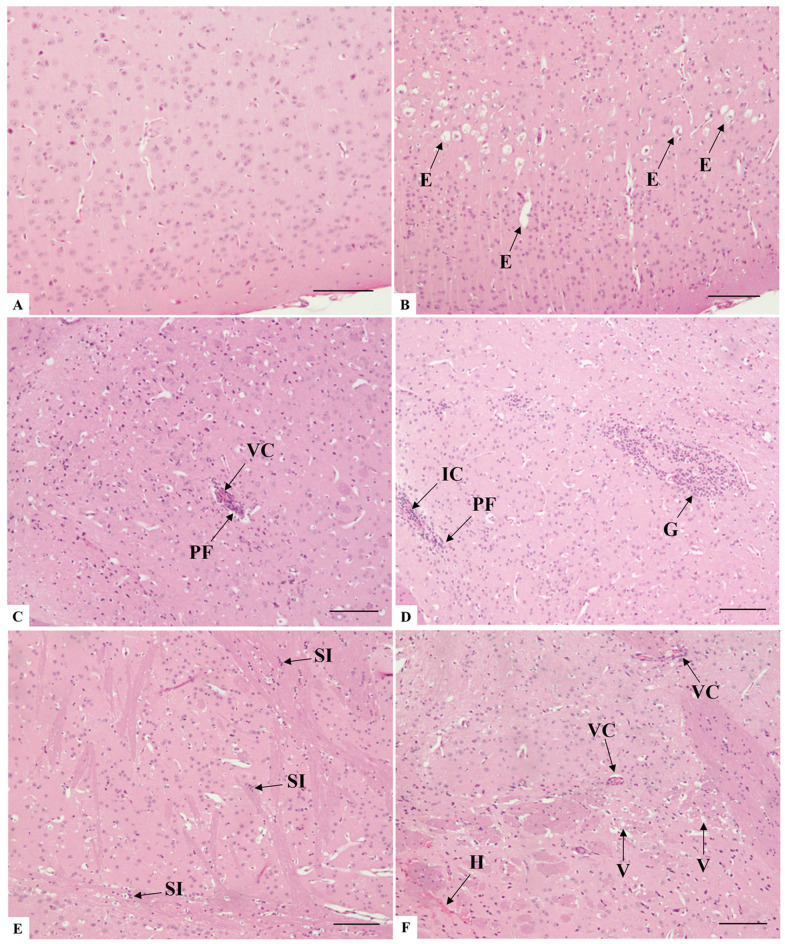
Histopathological analysis of brain sections of K18-hACE2 mice inoculated with PBS (**A**) and infected with SARS-CoV-2 (**B**–**F**). (**A**) Cerebral cortex area presenting no morphological alterations in neurons, neuropil, or vascular elements. (**B**) E: Edema. (**C**) PF: Perivascular inflammatory infiltrate; VC: Vascular congestion. Note the mild gliosis scenario throughout the field. (**D**) IC: Inflammatory cuffing; G: Gliosis; PF: Perivascular inflammatory infiltrate. (**E**) SI: Inflammation of striatum fibers. (**F**) H: Hemorrhage; V: Vacuolation of striatum fibers; VC: Vascular congestion. Bar: (**A**–**F**) 100 μm.

At seven days post-infection with SARS-CoV-2, analysis of brain histological sections revealed several notable changes, compared with the regular morphology observed in the negative control group mice (Figure 6A). The most significant alterations were observed in the cerebral cortex, whereas no morphological alterations were detected in the cerebellum sections, despite their analysis. There was evidence of edema in the brain tissue, characterized by swelling and cytoplasmic expansion around neurons (Figure 6B). However, the most frequent finding in the brain was the infiltration of mixed inflammatory cells in perivascular regions, indicating an ongoing inflammatory response (Figure 6C,D). It was not uncommon for the regions near the infiltration to exhibit mild to moderate gliosis (Figure 6C,D).

In the striatum, there was notable glial proliferation observed between bundles of myelinated fibers, within extensive eosinophilic plaques (Figure 6E,F). The extravasation of erythrocytes also occurred, sometimes coupled with blood stasis in small vessels, which suggests impaired blood flow (Figure 6F).

**Figure 7:**
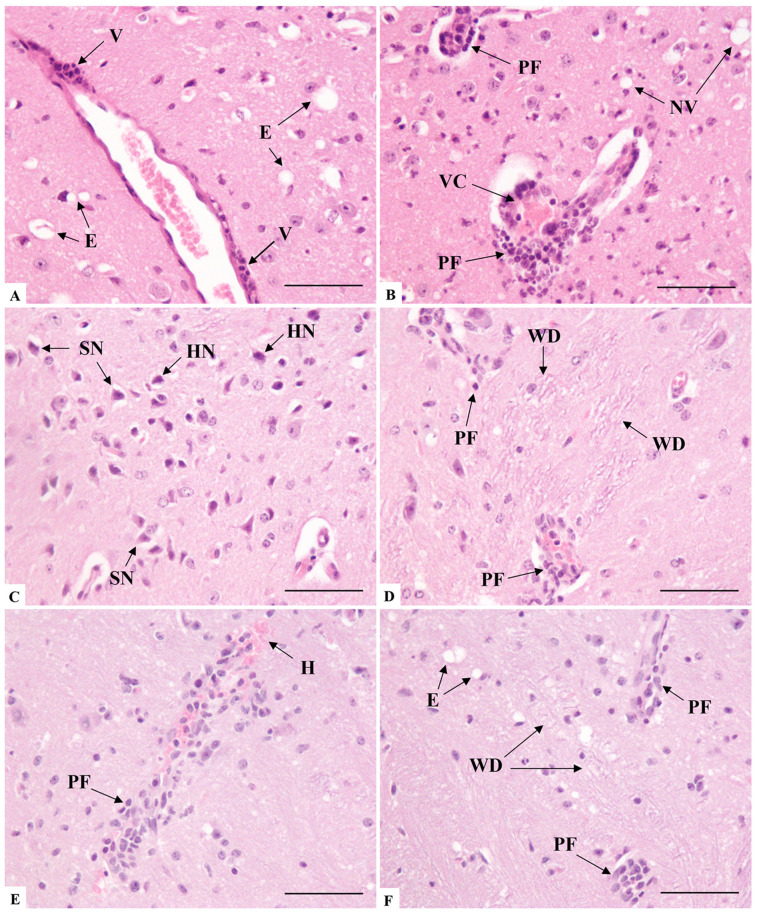
Histopathological analysis of brain sections of K18-hACE2 mice infected with SARS-CoV-2 (**A**–**D**). (**A**) E: Edema; V: Vasculitis. (**B**) NV: Neuropil vacuolation; PF: Perivascular inflammatory infiltrate; VC: Vascular congestion. (**C**) Note area of basophilic neuronal degeneration, presenting shrunken neurons (SN) and hyperchromatic nuclei (HN). (**D**) PF: Perivascular inflammatory infiltrate; WD: White matter demyelination. (**E**) H: Hemorrhage; PF: Perivascular inflammatory infiltrate. (**F**) E: Edema; PF: Perivascular inflammatory infiltrate; WD: White matter demyelination. Bar: (**A**–**F**) 50 μm.

The blood vessels were undoubtedly structures with a high degree of alteration, especially in the inflammatory component of endothelial infiltration (Figure 7A), which led to the distension of the Virchow’s space and involved the adjacent neuropil (Figure 7B). The occlusion of vessels, both by inflammatory cuffing and blood congestion (Figure 7B,D), often resulting in the mild extravasation of red blood cells (Figure 7E), was observed.

Regarding neuronal alterations, areas containing neurons with generalized nuclear hyperchromasia, central nucleoli, and pycnotic nuclei were widely seen (Figure 7C). In addition, the degeneration of white matter was observed, with demyelination and loss of axonal integrity (Figure 7C,D). In addition, the degeneration of white matter was observed, with demyelination and loss of axonal integrity, often accompanied by areas of edema, a recurring alteration in the analyses (Figure 7A,B,F).

### 3.6. Brain Ultrastructure

**Figure 8:**
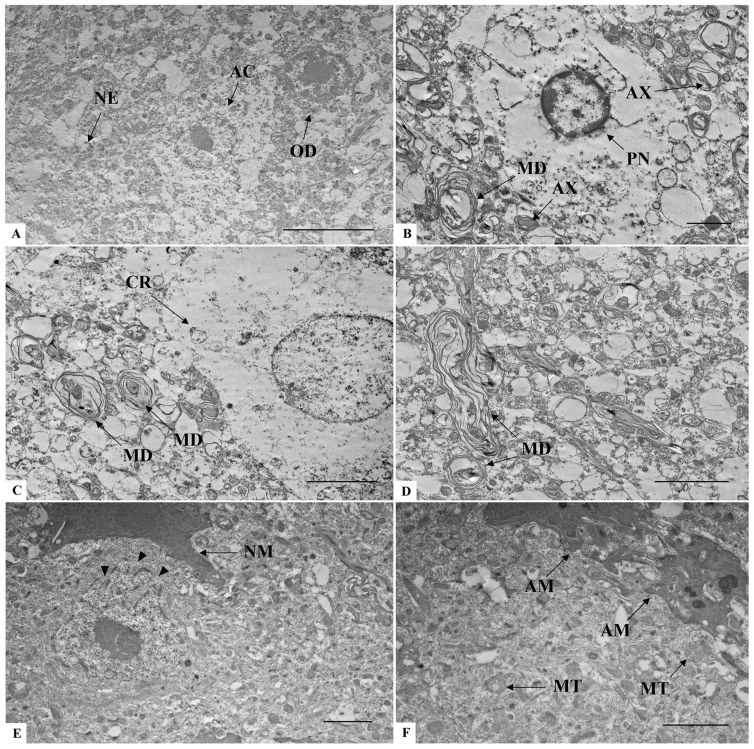
Ultrastructural analysis of brain sections of K18-hACE2 mice inoculated with PBS (**A**) and infected with SARS-CoV-2 (**B**–**F**). (**A**) AC: Astrocyte; NE: Neuropile; OD: Oligodendrocyte. (**B**) AX: Axonal detachment; MD: Multimyelin decompaction; PN: Pycnotic nucleus. (**C**) CR: Cytoplasmic rarefaction; MD: Multimyelin decompaction. (**D**) MD: Multimyelin decompaction. (**E**) Arrowhead: Nucleoplasmic reticulum; NM: Normal dark microglia. (**F**) AM: Activated dark microglia; MT: Mitochondria degeneration. Note the complex cytoplasmic extensions in the communication between two dark microglia. Bar: (**A**,**C**,**D**) 5 μm, (**B**,**E**,**F**) 2 μm.

In the negative control group (Figure 8A), the brain cells and their constituents presented well-preserved ultrastructural integrity. In the surrounding neuropil, no alterations were observed related to axons, dendrites, nor glial processes. In the infected mice, cell death was widely present, mostly characterized by shrunken cells with pyknotic nuclei (Figure 8B). Other cellular alterations included cytoplasmic rarefaction and nuclear anomalies in degenerating cells (Figure 8C). Myelin changes such as decompaction and multimyelination also occurred, including the formation of extensive myelin outfolds (Figure 8C,D). Another alteration was the presence of nuclear invaginations, connecting the cytoplasmic content with the nucleus (Figure 8E). Dark-activated microglia were also observed, next to a region with mitochondria degeneration (Figure 8F).

### 3.7. Gene Expression in Lung and Brain Tissues

**Figure 9:**
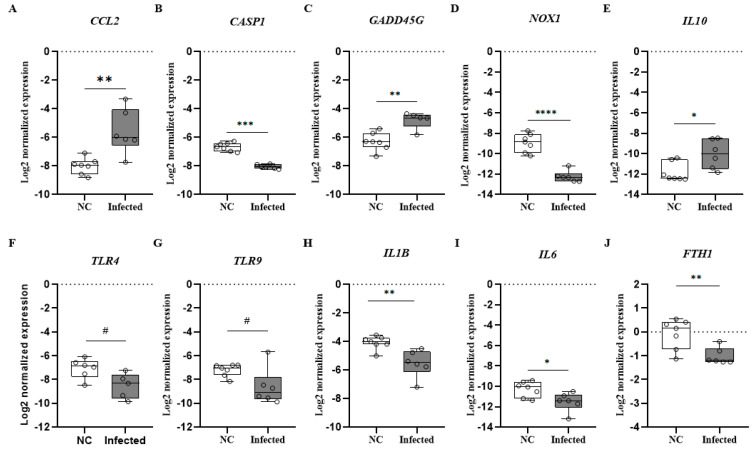
Levels cytokine/chemokine genes expression in lung tissue in K18-hACE2 mice infected with SARS-CoV-2. (**A**) *CCL2*; (**B**) *CASP1*; (**C**) *GADD45G*; (**D**) *NOX1*; (**E**) *IL10*; (**F**) *TLR4*; (**G**) *TRL9*; (**H**) *IL1B*; (**I**) *IL6*; (**J**) *FTH1*. Mann–Whitney test was applied to compare group mice negative control n = 7 (NC) and infected with SARS-CoV-2 n = 7. # *p* < 0.1, * *p* < 0.05, ** *p* < 0.01, *** *p* < 0.001, and **** *p* < 0.0001.

This analysis is derived from a subset of data previously included in a broader study conducted by our group that focused on the immunomodulatory effects of lactoferrin in the context of SARS-CoV-2 infection [22]. We conducted a reanalysis to explore significant differences between negative control (n = 7) and infected groups (n = 7) genic expression of inflammatory mediators in the lung and brain tissue of mice. Lung tissue gene expression analysis revealed a significant increase in *CCL2*, *GADD45G*, and *IL10* in the infected group, whereas *IL1B*, *IL6*, *CASP1*, *TLR4*, *TLR9*, and *FTH1* expression levels (Figure 9) were markedly reduced compared with the control group. The upregulation of *CCL2* suggests an active cellular recruitment process in response to infection (Figure 9A).

**Figure 10:**
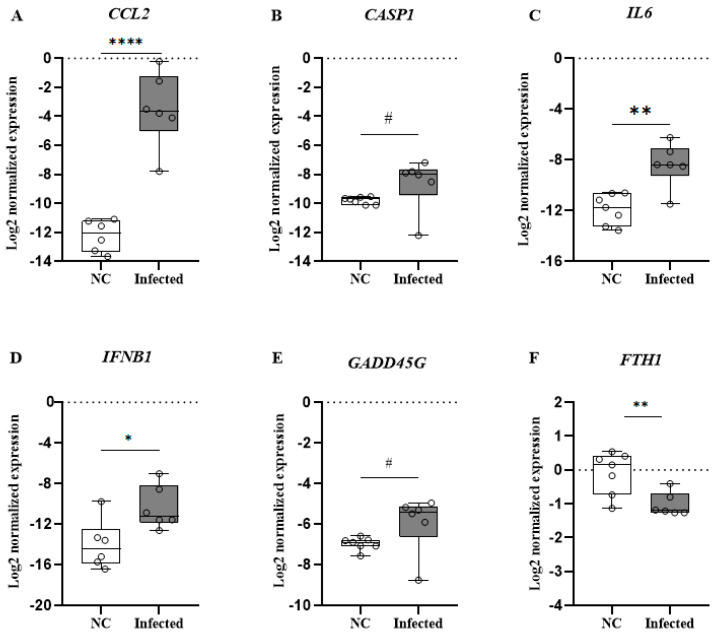
Levels cytokine/chemokine genes expression in brain tissue in K18-hACE2 mice infected with SARS-CoV-2. (**A**) *CCL2*; (**B**) *CASP1*; (**C**) *IL6*; (**D**) *IFNB1*; (**E**) *GADD45G*; (**F**) *FTH1*. Mann–Whitney test was applied to compare group mice negative control (NC) and infected with SARS-CoV-2. # *p* < 0.1, * *p* < 0.05, ** *p* < 0.01, and **** *p* < 0.0001.

Similarly, the increased expression of *GADD45G* suggests the activation of protective pathways aimed at mitigating tissue damage (Figure 9C). Notably, the significant elevation in *IL10* highlights the engagement of regulatory mechanisms that may help counterbalance the inflammatory response typically observed during SARS-CoV-2 infection (Figure 9E). In contrast, brain tissue was demonstrated to have a significant upregulation of *CCL2*, *CASP1*, *IL6*, *IFNB1*, and *GADD45G* in the infected group suggesting a robust neuroinflammatory response (Figure 10). Interestingly, the decrease in *FTH1* expression observed in both the lung and brain tissues of infected mice (Figure 9J and Figure 10F) may be indicative of altered iron metabolism and increased susceptibility to oxidative stress, factors that can further aggravate lung and neuronal injury.

## 4. Discussion

In COVID-19, pulmonary damage remains the most relevant aspect and is manifested through distinct morphological features. In this viral infection, the terminal portion of the airways is diffusely affected, and the alveoli are largely compromised [23]. In human cases and animal models of SARS-CoV-2 infection, including our own, hyperplasia of type II pneumocytes is a recurrent change, with indirect implications for lung physiology [18,24,25,26]. Although it can be part of a regenerative process in lung architecture, the imbalance associated with prolonged and inefficient type II pneumocytes hyperplasia can result in surfactant imbalance, inflammatory cytokine release, and fibrosis [27].

Disease severity is powered by an observed increase in the population circulating alveolar macrophages, together with SARS-CoV-2-induced high levels of CCL2 in the mice lung [28]. It was observed that CCL2, a chemokine involved in the recruitment of monocytic cells, is upregulated in infiltrating cells on the lungs of patients with COVID-19 and in bronchoalveolar lavage fluid in severe COVID-19, evidencing the critical role of lung macrophages in disease progression [29]. While essential for viral clearance and tissue repair, excessive macrophage activation can exacerbate lung injury and contribute to cytokine storm, leading to systemic complications [30]. These macrophages also stood out in interstitial pneumonia, a significant manifestation of severe COVID-19, characterized by inflammation and fibrosis affecting the interstitial tissue [31]. The major consequence of this infiltration is the alveolar septal thickening, which represents a significant histopathological change observed in severe cases of COVID-19 [26]. Our histopathological analysis showed that this thickening primarily resulted from a hypercellularity profile resulting from inflammatory cells infiltration and type II pneumocytes.

However, the accumulation of edema fluid in the interstitial spaces, along with vascular congestion, also contributes to the rearrangement of lung architecture. This process is exacerbated by endothelial dysfunction caused by SARS-CoV-2 infection, which increases vascular permeability and allows for the leakage of fluid and proteins into the alveolar walls [32]. It is important to emphasize that, throughout the ultrastructural analyses, morphological evidence of endothelial cell activation was observed.

SARS-CoV-2 infection triggers a cascade of histological changes, such as fibrin deposition, which play a pivotal role in the pathophysiology of severe COVID-19, contributing significantly to both inflammatory responses and thrombotic complications within the lungs [33]. This process leads to the formation of fibrin-rich microthrombi and thromboemboli, particularly in severe cases [34]. These thrombi not only obstruct pulmonary blood flow but also contribute to vascular damage and exacerbate respiratory compromise, by impairing gas exchange, the usual hypercoagulability state seen in COVID-19 [35]. Furthermore, fibrin deposition can result in the formation of hyaline membranes and similar structures on the alveolar epithelium, further compromising lung function [36,37].

Another relevant deposition component, widely observed during the analyses, was collagen, distributed both in the intercellular area and in the alveolar space. SARS-CoV-2 infection seems to influence collagen production, increasing its deposition compared with other causes of acute respiratory distress syndrome [38]. The recurrence of this alteration has also been demonstrated in other K18-hACE2 mouse models [39]. The exacerbated neutrophilic activity observed in the present study, along with the elevated collagen concentrations, could be associated with more severe outcomes of COVID-19, including implications for long COVID [40].

Furthermore, in the current study, regions adjacent to collagen deposition revealed double-membrane vesicles (DMVs) containing viral-like particles. Although additional confirmation tools would be required to definitively identify them, nonetheless, the presence of these structures, located within debris from an unidentified cell type, underscores their significance. These compartments are specialized intracellular structures formed during the replication of certain RNA viruses, including the *Coronoviridae* family, which serve as active sites for RNA synthesis and replication, providing a protected microenvironment for efficient viral replication [41]. Therefore, a more detailed investigation into the molecular mechanisms underlying DMV formation is essential, as it offers promising avenues for antiviral drug development aimed at disrupting viral replication.

Alveolar hemorrhage is another hemodynamic disorder that represents a critical pathological feature observed in severe cases of COVID-19 [42,43]. Patients experiencing severe alveolar hemorrhage are at heightened risk of developing complications such as acute respiratory distress syndrome and multi-organ failure, both associated with poor disease prognoses [44]. Although diffuse hemorrhage was not widely observed in our study, the vascular congested state and extensive extravasation of proteinaceous content suggest increased vascular permeability. This process is more closely related to hyper-inflammation, which could be observed throughout our histological analysis, and to dysregulation in cytokine release [45], rather than to the direct effects of viral replication in these cells [46]. However, the viral proteins of SARS-CoV-2 have been shown to influence the vascular permeability of endothelial cells when overexpressed [47,48].

The overall critical and advanced lung damage observed in mice seven days after SARS-CoV-2 infection reverberates in the lung gene expression profile. The increase in the regulatory cytokine IL-10 may play a role in protecting the lungs from the immune-mediated damage that has already occurred [49]. However, IL-10 may modulate immune responses in ways that hinder viral clearance, particularly in severe cases as this immunoregulatory effect, while protective against tissue damage, might also impair the development of effective antiviral response [49].

The upregulation of *GADD45G*, a gene associated with cellular stress, along with the downregulation of key pro-inflammatory immunological mediators such as *IL-1β*, *IL-6*, *CASP1*, *TLR4*, and *TLR9*, suggests that the significant pulmonary damage observed at the histological and cellular levels may suppress innate immune response processes related to these genes, including TLR, NF-κB, and inflammasome activation [50]. This pattern aligns with other immune evasion mechanisms observed in SARS-CoV-2 infection, including the active inhibition of type I interferon (IFN-I) production and signaling by viral proteins, which facilitates viral persistence and occurs alongside alterations in the immunoregulatory cytokine environment, such as the upregulation of IL-10 expression [51].

A similar profile was observed in a study involving K18-hACE2 mice that were intranasally infected with SARS-CoV-2, resulting in severe lung inflammation. In this study, mice exhibited a typical cytokine storm in the lungs at two days post-infection; however, by seven days post-infection, there was a downregulation of chemokines and pro-inflammatory cytokines, coinciding with impaired pulmonary function [52]. Conversely, understanding the inflammatory processes underlying milder SARS-CoV-2 infection in K18-hACE2 mice underscores the importance of coordinated immune regulation in disease resolution, and highlights the therapeutic potential of targeted immunomodulation to mitigate disease severity in COVID-19 [53].

Beyond the pulmonary effects, neurological symptoms resulting from SARS-CoV-2 infection are extensive, including headache, brain fog, vertigo, loss of taste or smell, and, rarely, seizures, strokes, and movement disorders [6,54,55,56]. Any of these consequences are a source of concern, especially regarding long-term impairment [57,58]. However, most studies focus on conventional magnetic resonance imaging, which enables only a narrow range of lesion visualization, while analyses at the morphological and ultrastructural level of brain tissue are scarcer [59,60,61,62].

On the morphological scope, most studies focus on histopathological analyses, and alterations such as astrogliosis, microgliosis, vascular injury, and neuroinflammation are frequently observed in the K18-hACE model [24,63,64,65,66]. In terms of ultrastructural changes, the presence of neurons with vacuolated cytoplasm [64], neuronal chromatolysis, and pyknotic degeneration was reported [17].

The death of neural cells has been observed in human cases of SARS-CoV-2 infection [67,68] and, in our ultrastructural analysis, the cellular damage was mostly characterized by the presence of pyknotic nuclei and cytoplasmic rarefaction. Similar neuronal findings were seen in the hACE2-transgenic mice [64], which have proven to be an important model for understanding the effects of SARS-CoV-2 infection in the brain tissue.

Other neuron-related changes identified in the present study include the deeply invaginated nuclear membranes resulting in a novel structure, known as the nucleoplasmic reticulum, which increases the communication between nucleus and cytoplasm [69]. This structure was found in the post mortem human brain tissue of Alzheimer’s disease cases and is related to neurodegeneration [70]. Although its correlation with COVID-19 is not fully understood, the intense nucleocytoplasmic trafficking in SARS-CoV-2 [71,72,73,74] might be facilitated by the enhanced crosstalk promoted by the nucleoplasmic reticulum.

Regarding the glial cells, oligodendrocytes and Schwann cells are responsible for axon myelination, in which a multi-layered myelin sheath is spirally wrapped around axons [75]. The well-maintenance of this sheath is essential for adequate impulse conduction and axonal support [76,77]. Both the loss of myelin and changes in its structure, including the decompaction and formation of outfolds, are known to be associated with neurological diseases [78,79,80,81].

In SARS-CoV-2 infection studies, brain findings documented myelin alterations in those patients [59,82,83,84,85,86]. Subsequently, the malformation and degradation of myelin have been described as causes of neurological complications in COVID-19 and are associated to several origins, such as immunomodulatory response, direct damage of myelin-producing cells, and impaired cerebral blood flow [87,88,89].

The gene expression profile in mice brain tissue was characterized by an upregulation of pro-inflammatory responses, likely driven by microglia, contributing to the dysregulation of neural functions upon SARS-CoV-2 infection. Increased levels of NF-kB-induced cytokines such as IL6 and CCL2, together with IFNB1, suggest the activation of resident macrophages and microglia in the brains of these mice [90,91]. Additionally, elevated CASP1 levels indicate the activation of the inflammasome pathway, which may facilitate the induction of pro-inflammatory processes and tissue damage [92]. In contrast, the significant reduction in *FHT1* expression may be indicative of ferritinophagy in microglia, due to altered iron metabolism and increased susceptibility to ferroptosis, oxidative stress, and inflammation, factors that can further aggravate neuronal injury [93].

Whether in glial or neurons cells, mitochondrial alterations might lead to irreversible damage, interrupting vital processes, and viruses are known to “hijack” the functioning of mitochondria, which leads to enhanced infection and impairment of host immune response [94,95].

In vitro studies described ultrastructural changes related to mitochondria during SARS-CoV-2 infection including uncommon morphology and aggregates in certain areas of the cell [96,97]. Moreover, the mitochondria injury in SARS-CoV-2 brain infection appears to be closely linked to neurological symptoms in so-called long COVID, notably brain fog [98,99,100,101], which emphasizes the importance of this interaction to COVID-19 pathogenesis and to the future development of clinical approaches [102].

## 5. Conclusions

In summary, our findings provide insights into the morphological and transcriptional changes associated with SARS-CoV-2 infection, particularly the complex interplay between inflammation and hemodynamic disorders in the lungs and brain. These findings underscore the necessity for further research into the long-term effects of COVID-19, especially in relation to neurological sequelae and post-viral syndromes.

## Figures and Tables

**Table 1:** Clinical signs monitoring data of K18-hACE2 mice infected with SARS-CoV-2.

Clinical Signs/ Days Post-Infection (dpi)		Mice Identification																				
		A	B	C	D	E	F	G	H	I	J	K	L	M	N	O	P	Q	R	S	T	U
Piloerection	5 dpi																					
	6 dpi													X		X	X	X	X			
	7 dpi													X		X	X	*	X	X	X	X
Arched back	5 dpi												X									
	6 dpi								X	X	X		X ^e^	X	X ^e^	X	X	X	X	X	X	X
	7 dpi								X	X	X		-	X	-	X	X	*	X	X	X	X
Respiratory disfunction	5 dpi												X									
	6 dpi	X			X				X	X	X		X ^e^		X ^e^			X	X			
	7 dpi	X		X	X	X	X	X	X	X	X		-	X	-			*				
Mobility impairment	5 dpi																					
	6 dpi												X ^e^		X ^e^							
	7 dpi	X		X	X	X	X	X	X	X	X		-	X	-	X	X	*	X	X	X	X
Occular discharge	5 dpi												X									
	6 dpi	X			X				X				X ^e^		X ^e^					X	X	
	7 dpi	X		X	X	X	X	X	X				-		-	X	X	*	X	X	X	X

dpi: days post-infection; ^e^: humane endpoint; *: found dead.

## Data Availability

The original contributions presented in this study are included in the article. Further inquiries can be directed to the corresponding authors.
